# Evolution trends over three decades of HIV infection late diagnosis: the experience of a Portuguese cohort of 705 HIV-infected patients

**DOI:** 10.7448/IAS.17.4.19688

**Published:** 2014-11-02

**Authors:** Ana Claudia Miranda, Virginia Moneti, Pedro Brogueira, Susana Peres, Teresa Baptista, Isabel Aldir, Fernando Ventura, Fernando Borges, Kamal Mansinho

**Affiliations:** Serviço de Infecciologia e Medicina Tropical, Hospital Egas Moniz Centro Hospitalar de Lisboa Ocidental, Lisbon, Portugal

## Abstract

**Introduction:**

Late HIV diagnosis is common and associated with an increased risk of clinical progression, blunted immune response on antiretroviral (ARV) therapy and higher risk of drug toxicity. Across Europe, more than a third of patients are diagnosed late and consequently delay medical care. European Consensus definition group identify as late presentation (LP) persons, presenting for care, with a CD4 count below 350 cell/mm^3^ or presenting with AIDS-defining event, regardless of CD4 cell count. Additionally, advanced HIV disease (AD) is defined by a CD4 count below 200 cell/mm^3^ or an AIDS defining condition in persons presenting to care.

**Materials and Methods:**

Retrospective observational study of a cohort of 705 HIV-infected patients diagnosed between 1986 and 2014 and medically followed at an Infectious Diseases Service in Lisbon.

**Objectives:**

Evaluate LP rate evolution in the last three decades (10-year time intervals considered: 1986–1995; 1996–2005; 2006–2014); compare clinic, immunologic, virologic and therapeutic response over time. Identify main reasons responsible for late HIV diagnosis in order to promote optimized intervention strategies. SPSS version 20.0 was used for statistical analysis.

**Results:**

Study included 705 patients HIV diagnosed during 3 time intervals: group A n=82 [1986–1995]; group B n=332 [1996–2005]; group C n=291 [2006–2014]. Demographic and epidemiological characterization revealed (A vs B vs C): male predominance of 79% vs 66% vs 66%; mean age at diagnosis 30 vs 36 vs 42 years; Portugal (82% vs 70% vs 58%) and Africa (13% vs 23% vs 29%) as the main places of birth; transmission by heterosexual contact in 21% vs 47% vs 62%, MSM in 21% vs 15% vs 23% and IVDU in 57% vs 35% vs 13%. Mean CD4 at diagnosis was 362 vs 344 vs 377 cell/mm^3^. Considering the time intervals, LP was found in 52% vs 56% vs 52% of patients and AD in 31% vs 38% vs 35%, respectively. At first health care encounter, 46% vs 43% vs 39% of individuals presented with AIDS. Over follow up, the vast majority initiated ARV (95% vs 98% vs 84%) and mean CD4 at that time was 254 vs 282 vs 250 cell/mm^3^. The last immunologic and virologic determination available registered mean CD4 of 657 vs 644 vs 584 cell/mm^3^ and undetectable HIV plasma RNA in 92% vs 84% vs 82% of treated patients.

**Conclusions:**

This study evidenced a maintained LP rate, slightly above 50% in each of the three analyzed last decades, and one-third of patients presented AD at HIV diagnosis. At initial health care contact, nearly 40% of individuals met AIDS clinical or immunological criteria.

